# Supplementary material for: Robust cortical encoding of 3D tongue shape during feeding in macaques
Source: Nat Commun. 2023 May 24;14:2991. doi: 10.1038/s41467-023-38586-3 (PMC10209084; doi:10.1038/s41467-023-38586-3)
Supplement: Supplementary file 1 — Supplementary Information [file 41467_2023_38586_MOESM1_ESM.pdf]

Supplementary Information

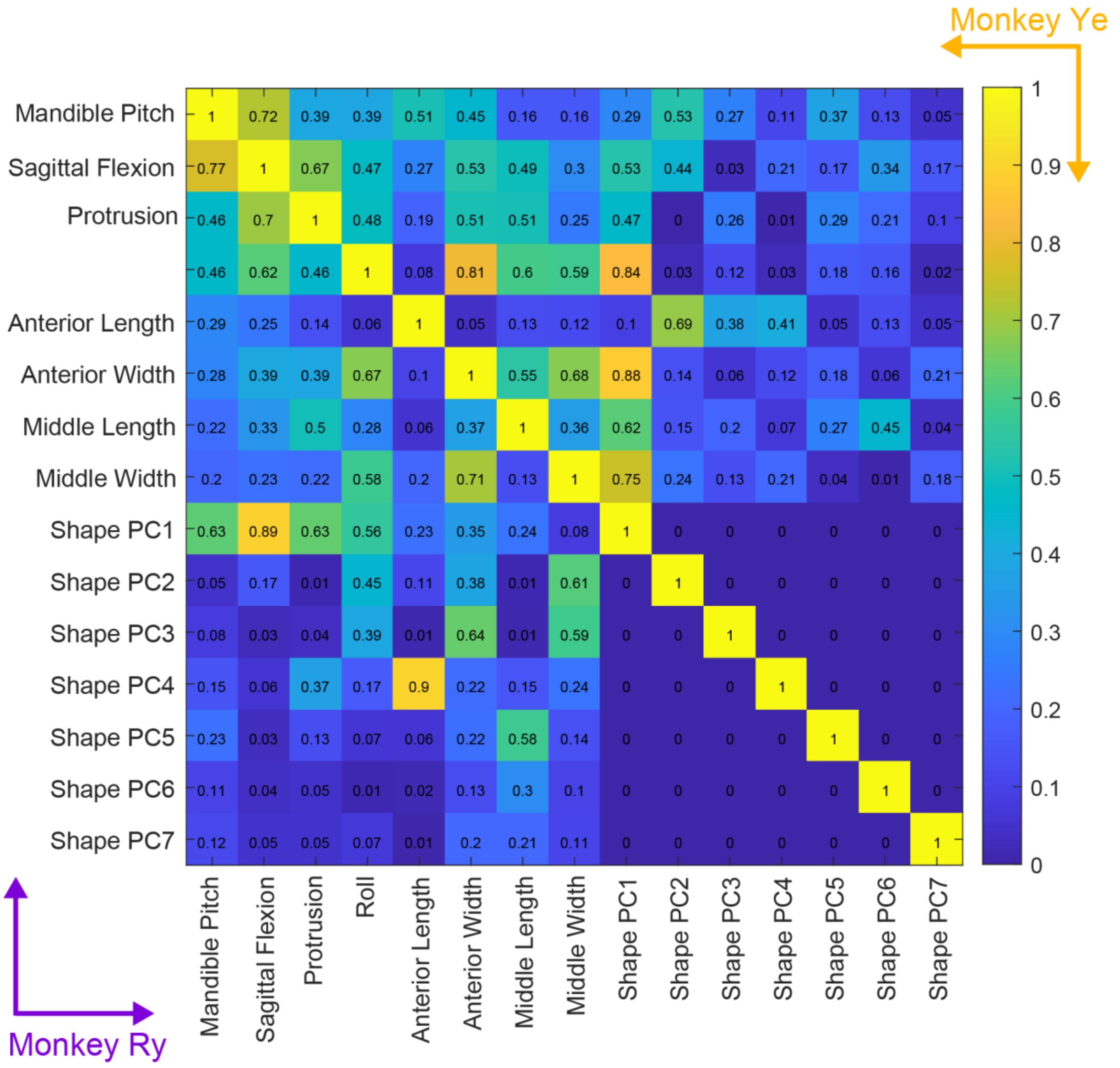

Supplementary Figure 1 | Correlations (Pearson's *r*) between kinematic variables. Correlations were computed on the complete kinematics datasets (test and train), and the absolute value of the correlation coefficients was taken to aid in comparison. The lower left half is Monkey Ry and the upper right half is Monkey Ye. Mandibular pitch was not a decoded variable in this study, but constitutes the biomechanical framework for feeding, and thus was included in this matrix. Note that the values for the shape PC variables are expected, as orthogonality (zero-correlation) is definitional to the construction of principal components.

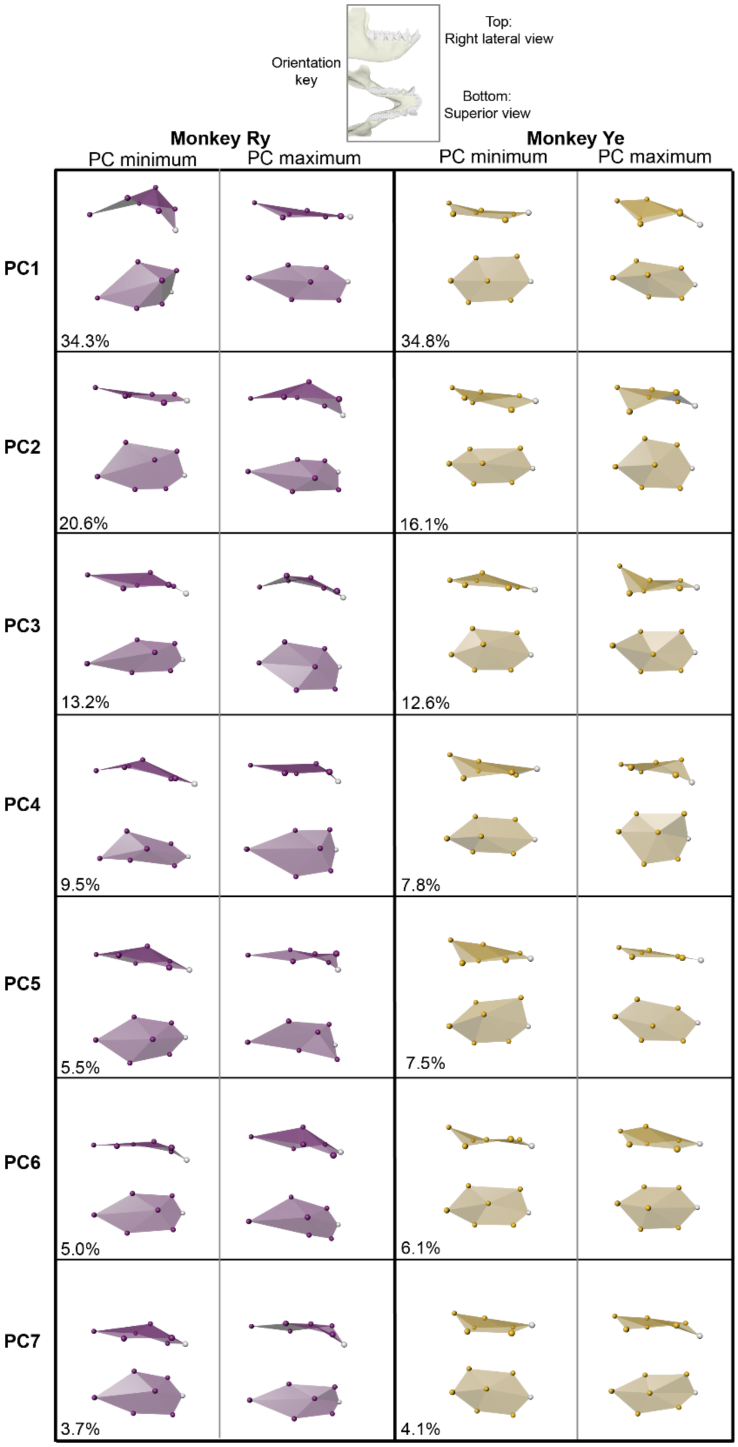

Supplementary Figure 2 | Visual representation of principal components of tongue shape. Each box contains a 3D rendering (top, right lateral view; bottom, superior view) of the 7 tongue markers in reconstructed poses (connected by a polygonal mesh to aid in visualization). The poses represent tongue shape at maximum (right) and minimum (left) observed values for a given PC in the entire dataset. In all images, the white sphere is the tongue tip and anterior is to the right.

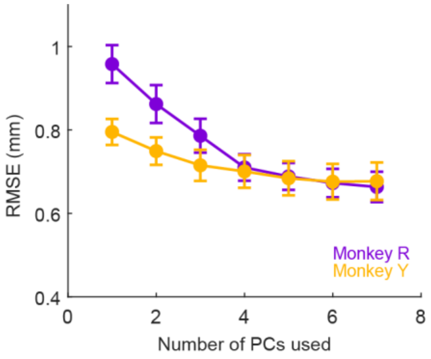

Supplementary Figure 3 | Root-mean-square decoder error by number of principal components used in shape reconstruction. We reconstructed the 3D Procrustes shape-space positions of all 7 tongue markers from a progressively larger number of independently decoded PC scores. X-axis position indicates the number of decoded PCs (in order of % variance explained) used to reconstruct the 3D marker positions. Values are the 3D root-mean-square error (RMSE) of the reconstructed markers and error bars depict standard deviation across 6 folds of cross-validation. Unsurprisingly, as additional decoded PCs are added to the reconstruction, the error of the reconstruction decreases. See Supplementary Table 1 for additional decoding information.

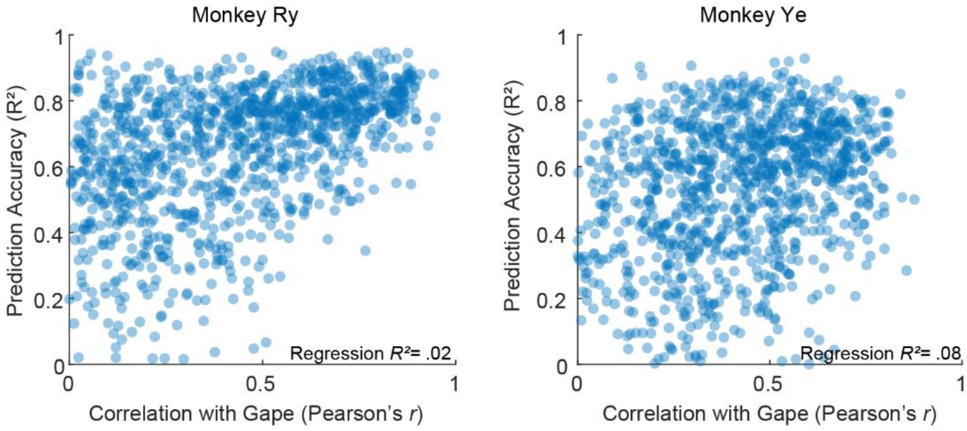

Supplementary Figure 4 | Relationship between decoding accuracy and correlation with gape (jaw pitch). Each circle depicts the tongue-jaw correlation (x-axis) and decoding accuracy (y-axis) of a single decoded variable, for a randomly drawn 3-s segment of test data. A total of 50 random segments were drawn. The relatively low R^2 values (via linear regression) indicate that a large proportion of the tongue decoding performance cannot be accounted for by correlations with the jaw. See Supplementary Table 1 for additional decoding information and *XROMM Data Processing* section of Methods for details on mandible motion reconstruction and jaw pitch calculation.

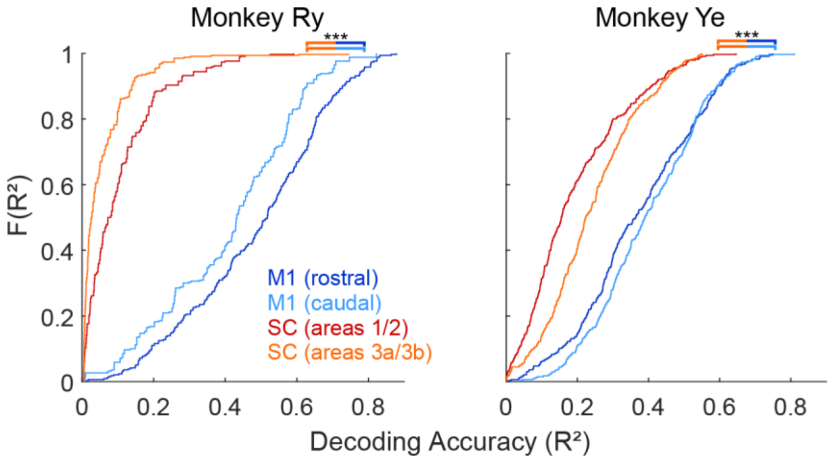

Supplementary Figure 5 | Comparison of decoding accuracy from different OSMcx regions. Cumulative distribution functions depict the accuracy (R^2) of tongue kinematics decoded from neural responses in multiple cortical areas in two monkeys. Data are pooled trial-level R^2 values for all kinematic variables for 18 held-out test trials (6 folds). Both rostral and caudal M1 significantly outperformed the two SC regions ($P>0.05$, two-sided Wilcoxon Rank Sum Test), and there was little difference between the two arrays in the same region (M1 or SC).

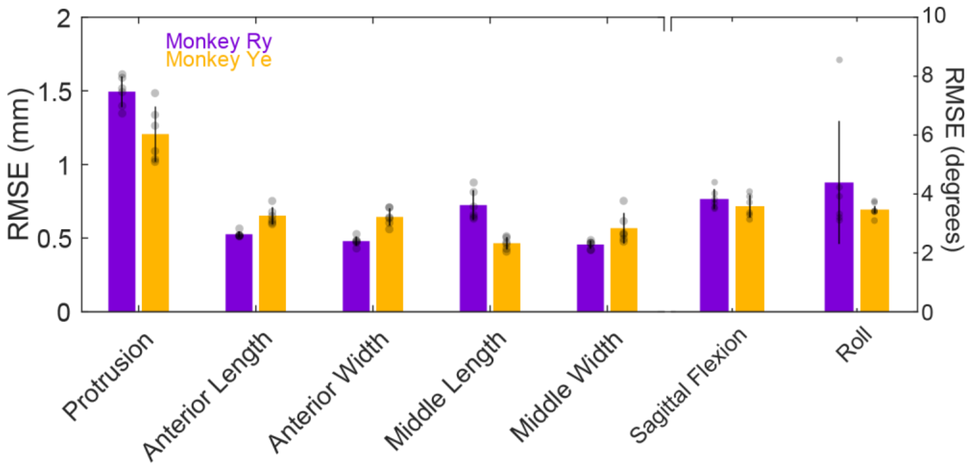

Supplementary Figure 6 | Root-mean-square decoder error of tongue kinematics. Values are the root-mean-square error (RMSE) of each decoded kinematic variable (units: left, mm; right, degrees) and error bars depict ±1 standard deviation across the folds of cross-validation (n=6). See Supplementary Table 1 for additional decoding information.

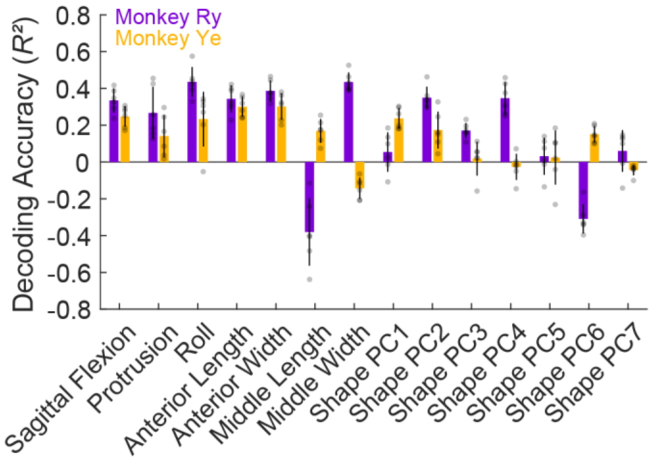

Supplementary Figure 7 | Kalman filter decoding performance. Values are decoding accuracy (R^2), by variable, for both subjects. Each bar represents the mean Kalman filter decoding accuracy for that variable across the folds of cross-validation (n=6; input data was identical to that used in the LSTM decoding analysis). Error bars represent ± 1 standard deviation.

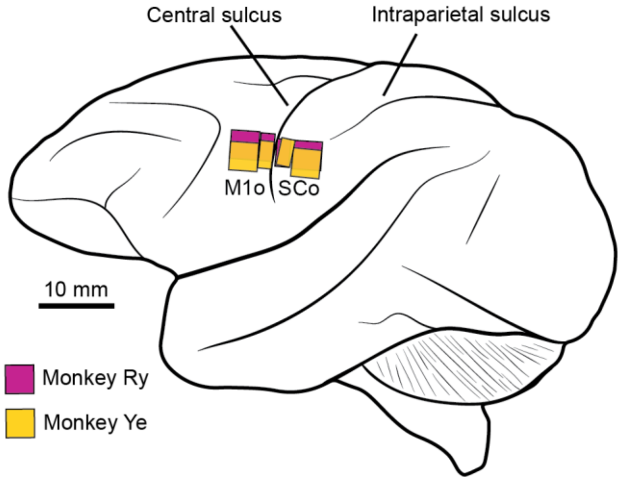

Supplementary Figure 8 | Microelectrode array locations in each subject. Squares are Utah arrays and rectangles are floating microelectrode arrays. Drawing is to scale and array locations were taken from surgical photographs.

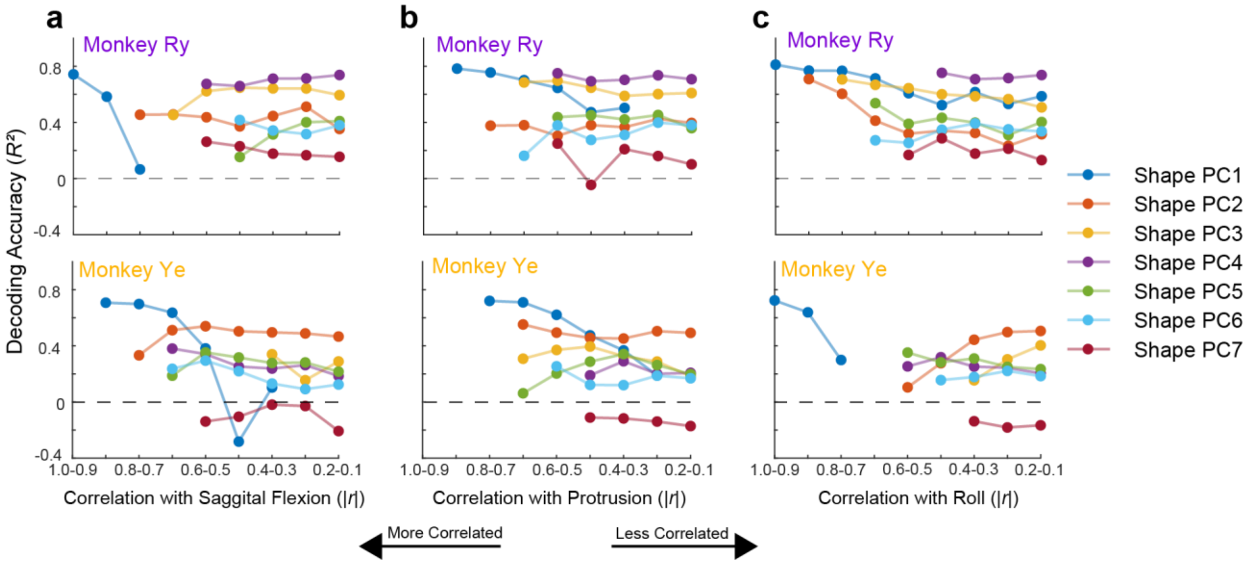

Supplementary Figure 9 | Shape decoding during periods of weak kinematic correlation. Values are mean decoding accuracy (R^2), by shape variable, of randomly drawn 2-s segments (n=15-50 segments per circle) of test data where the Pearson correlation of the shape variable with the given kinematic variable (Sagittal flexion, **a**; protrusion, **b**; roll, **c**), was within the specified range. Absences of circles indicate that there were fewer than 15 segments with a shape-kinematic correlation within the given range for that variable.

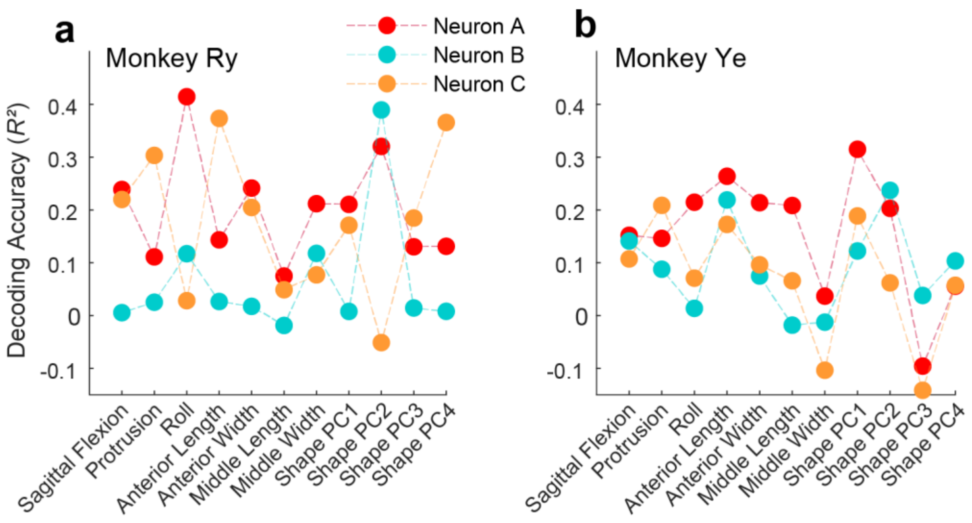

Supplementary Figure 10 | Decoding performance of individual cortical neurons. Values are decoding accuracy (R^2), by variable, for three high-performing M1 neurons from Monkey Ry (a) and Monkey Ye (b). Each circle represents the decoding accuracy for that variable across 3 folds of cross-validation.

Figure Panel	Subject	Training set size	Data counts	Data detail	Error Bars
**1d,1e**	Ry	24 trials (6-10 sec duration)	Representative trial	2 s / 400 frames	-
**2a, 3a**	Ry	“”	Sub-sequence of representative test (held out) trial	4.4 s / 880 frames	-
**2b, 3b**	Ry+Ye	“”	6 test folds	Each test fold comprises 4 test trials with durations of 6-10 s	1 standard deviation
**4a**	Ry+Ye	“”	6 test folds	“”	**-**
**4b**	Ry+Ye	“”	5 iterations for each ensemble size, 3 test folds per iteration	“”	1 standard deviation
**4c**	Ry+Ye	“”	40 iterations/neurons, 3 test folds per neuron	“”	

Supplementary Table 1 | Figure data information.

Figure Panel	Number of hidden units	Solver	Initial Learning Rate	Epochs	Regularization (L1, L2)
**2a,b, 3a,b 4a,b**	200	Adam optimizer	0.001	200	None
**4b (ensemble size of 1), 4c**	80	Adam optimizer	0.001	125	None

Supplementary Table 2 | LSTM network parameters.
