# Supplementary material for: Robust cortical encoding of 3D tongue shape during feeding in macaques
Source: Nat Commun. 2023 May 24;14:2991. doi: 10.1038/s41467-023-38586-3 (PMC10209084; doi:10.1038/s41467-023-38586-3)
Supplement: Supplementary file 3 — Description of Additional Supplementary Files [file 41467_2023_38586_MOESM3_ESM.pdf]

File name: Supplementary Movie 1

Description: Reconstruction of tongue shape principal components. Rendering generated with Autodesk Maya 2022.

File name: Supplementary Movie 2

Description: Real (black) and decoded (red) tongue shape from a representative test trial from monkey Ry. Marker positions are reconstructed from the 7 independently decoded shape principal components. Decoder input was the time-varying firing rates of 100 primary motor cortex neurons. Mandible is rendered for orientation purposes only, as shape-based marker positions are coordinate system independent. Playback speed is .25x real time. Rendering generated with Autodesk Maya 2022.
